# Long non‐coding RNA ZEB2‐AS1 promotes the proliferation, metastasis and epithelial mesenchymal transition in triple‐negative breast cancer by epigenetically activating ZEB2

**DOI:** 10.1111/jcmm.14213

**Published:** 2019-03-01

**Authors:** Guoxin Zhang, Hongli Li, Ruimei Sun, Peirui Li, Zhiyi Yang, Yuanyuan Liu, Zhaoyan Wang, Yuling Yang, Chonggao Yin

**Affiliations:** ^1^ College of Biological Science and Technology Weifang Medical University Weifang China; ^2^ Medicine Research Center Weifang Medical University Weifang China; ^3^ Affiliated Hospital, Weifang Medical University Weifang China; ^4^ Department of Pathology Weifang Medical University Weifang China; ^5^ College of Nursing Weifang Medical University Weifang China

**Keywords:** cytoskeleton rearrangement, epithelial mesenchymal transition (EMT), LncRNA‐ZEB2‐AS1, triple‐negative breast cancer

## Abstract

The triple‐negative breast cancer is the most malignant type of breast cancer. Its pathogenesis and prognosis remain poor despite the significant advances in breast cancer diagnosis and therapy. Meanwhile, long noncoding RNAs (LncRNAs) play a pivotal role in the progression of malignant tumors. In this study, we found that LncRNA‐ZEB2‐AS1 was dramatically up‐regulated in our breast cancer specimens and cells (MDA231), especially in metastatic tumor specimens and highly invasive cells, and high lncRNA‐ZEB2‐AS1 expression is associated with clinicopathologic features and short survival of breast cancer patients. LncRNA‐ZEB2‐AS1 promotes the proliferation and metastasis of MDA231 cells in SCID mice. Thus, it is regarded as an oncogene in triple‐negative breast cancer. It is mainly endo‐nuclear and situated near ZEB2, positively regulating ZEB2 expression and activating the epithelial mesenchymal transition via the PI3K/Akt/GSK3β/Zeb2 signaling pathway. Meanwhile, EGF‐induced F‐actin polymerization in MDA231 cells can be suppressed by reducing lncRNA‐ZEB2‐AS1 expression. The migration and invasion of triple‐negative breast cancer can be altered through cytoskeleton rearrangement. In summary, we demonstrated that lncRNA‐ZEB2‐AS1 is an important factor affecting the development of triple‐negative breast cancer and thus a potential oncogene target.

## 1 INTRODUCTION

Breast cancer is a malignancy with high death rate; that is, individuals with this disease, especially women, have a five‐year overall survival rate of less than 15%.^1^ Having high metastasis and recurrence rates, breast cancer is difficult to treat through prophase clinical diagnosis. Although major breakthroughs in clinical treatment methods have been achieved for breast cancer, approximately 100 in 100 000 women aged 55‐69 years are expected to contract the disease by 2021.^2^ Inhibiting the invasion and metastasis of malignant tumors is the most in clinical treatment, and it is also a hot and difficult issue of current research.

Many previous studies confirmed that epithelial mesenchymal transition (EMT) is one of the main mechanisms that cause the dispersion of malignant tumors.^3^ EMT triggers a variety of biological changes in normal mammary epithelial cells, which eventually obtains the characteristics of mesenchymal cells. This effect enhances the metastasis capacity, invasiveness and resistance of cancer cells, thereby preventing apoptosis and promoting the production of extracellular matrix components.^4^ The occurrence of EMT is related to a variety of molecular mechanisms and signaling pathways.^5^ Its main function is the reduction of intercellular adhesion and E‐cadherin, mesenchymal vimentin, and N‐cadherin expression. Moreover, many transcriptional factors, including snail and ZEB, have crucial roles in EMT‐induced processes.^6^ The ZEB family (ZEB1 and ZEB2) is closely related to the EMT‐related markers of malignant tumors.^7^ However, the regulatory role of lncRNA‐ZEB2‐AS1 in ZEB2 expression in breast cancer remains unreported.

LncRNA is a kind of regulatory RNA that has a transcriptional length of more than 200 nucleotides and has no protein coding ability.^8^ LncRNA plays a vital role in malignant tumor development, especially in apoptosis, proliferation, and invasion.^9^, ^10^, ^11^ Abnormal lncRNA expression is observed in malignant tumors, such as gastric cancer, hepatocellular carcinoma (HCC), and glioma.^12^, ^13^, ^14^ Moreover, lncRNA is a competitive endogenous RNA (ceRNAs) that regulates miRNA.^15^, ^16^ Despite the significant role lncRNA in malignant tumor development, its regulatory functions and molecular mechanisms remain poorly understood.

In this study, we found that abnormal lncRNA‐ZEB2‐AS1 expression is associated with patient survival and prognosis and controls ZEB2 expression. In the MDA231 cells, LncRNA‐ZEB2‐AS1 promoted the proliferation and metastasis of tumor cells and triggered EMT via the PI3K/Akt/GSK3β/Zeb2 signaling pathway and F‐actin polymerization.

## 2 MATERIAL AND METHODS

### 2.1 Clinical specimens

We obtained breast cancer specimens (BC) and adjacent no tumor (ANT) specimens from the Affiliated Hospital of Weifang Medical University from 2011 to 2016. All the specimens were frozen to −150°C. None of the specimens received radiotherapy or chemotherapy before surgery. Informed consent was obtained from each patient. The study was approved by the Research Ethics Committee of Weifang Medical University.

### 2.2 Cell culture

MCF‐10A, T47D, MDA‐MB‐435 (MDA435), MCF‐7, and MDA‐MB‐231(MDA231) were acquired from ATCC (USA). The cells were cultured in minimum essential medium with 10% fetal bovine serum (FBS) at 37℃ under 5% CO_2_ atmosphere.

### 2.3 Plasmid construction and cell transfection

MDA231 cells (2 × 10^5^) were planted in six‐well plates and left overnight. We used Lipofectamine 2000 to transfect the MDA231 cells in accordance with the manufacturer protocol Lnc‐ZEB2‐AS1‐RNAi, and si‐NC plasmids were constructed by GeneChem company (shanghai, China) Sequences were provided in Table S1. Stable transfected cells were maintained with 300 μg/mL G418 for 14 days.

### 2.4 Wound healing assay

Approximately 2 × 10^5^ breast cancer cells were planted onto six‐well plates and then left overnight. Wounds were created with a 10 μL pipette tip. Complete medium including 1% FBS was added to the plates with or without EGF. The entire setup was photographed after 36 hours.

### 2.5 Cell proliferation assays

For the cell counting kit 8 (CCK‐8; Solarbio, Beijing) assays, approximately 2 × 10^3^ cells were seeded in a 96‐well plate. The proliferation capacities of the cells cultured for 24, 48, 72, 96, and 120 hours were tested by the CCK‐8 assays. The cell growth curves were plotted by using the absorbance value at each time point.

### 2.6 Transwell assay

MDA231 cells (1 × 10^5^) were counted and planted in serum‐free essential medium in the upper chamber. Matrigel (Corning) was used for this step. The bottom chamber was added into essential medium with EGF. The cells were incubated for 24 hours at 37°C. The membranes of the MDA231 cells were fixed and stained, photographed, and counted.

### 2.7 Western blot

Protein expression was examined as described previously.^17^ The following antibodies were employed: ZEB2 (Santa Cruz, 1:500), N‐cadherin (Cell Signaling Technology, CST, 1:500), E‐cadherin (CST, 1:500), vimentin (CST, 1:1000), GSK3β (CST, 1:500), p‐GSK3β^Ser9^ (CST, 1:500), p‐Akt ^Ser473^ (CST, 1:500), Akt (CST, 1:500), p‐LIMK^Thr508^ (CST, 1:500), p‐cofilin^Ser3^ (CST, 1:500), β‐actin (CST, 1:2000), HRP‐conjugated anti‐mouse IgG and anti‐rabbit IgG antibody (CST, 1:5000). All experiments were performed in triplicate.

### 2.8 Quantitative real‐time PCR

We performed Quantitative real‐time PCR (QRT‐PCR) to define the relative level of lncRNA‐ZEB2‐AS1. GAPDH levels were used for normalization. We used TRIzol to extract RNA from fresh breast cancer specimens and cells. Then, cDNA was synthesized with the total RNA by using an M‐MLV reverse transcriptase kit (Promega, USA). Relative mRNA expression was normalized through the 2 ^−ΔΔCT^ method. QRT‐PCR was carried out using an Applied Biosystems 7500.

### 2.9 Cellular F‐actin measurement

MDA231 cells were immobilized with 4% paraformaldehyde with PBS. The cells were then washed three times before they were blocked with a buffer containing goat serum for 45 minutes. The MDA231 cells were stained with Phalloidin (Fluorescein isothiocyanate) FITC for 1 hours, washed three times, and covered with fluorescence decay resistant sealing tablets. Data processing was performed as described previously.^18^

### 2.10 Immunofluorescence

The MDA231 cells were fixed with 4% paraformaldehyde for 25 min. The cells were permeabilized with 0.1% Triton X‐100 and blocked with goat serum for 45 min at 37°C. The primary antibodies of relation were incubated at 4°C overnight. FITC, Cy3‐labeled goat anti‐rabbit IgG secondary antibodies, and DAPI were used.

### 2.11 Animal studies

The Server Combined Immune‐deficiency (SCID) mice were provided by the Animal Care and Use Committee of Wei Fang Medical University. Si‐NC/MDA231 and MDA231/si‐ZEB2‐AS1 cells (2 × 10^6^) were injected into the oxter of each female SCID mouse (n = 10). When the xenografts became evident, tumor volume was measured. After 7 weeks, metastasis in the lung tissues was examined by HE staining.

### 2.12 Statistical analysis

Data were analyzed by SPSS v16.0. All values were expressed as mean ± SD. The results were analyzed with T test or ANOVA. Χ^2^‐test was used for the analysis of the connection between lncRNA‐ZEB2‐AS1 and the clinicopathologic features. *P* < 0.05 was considered significant in all cases

## 3 RESULTS

### 3.1 Up‐regulation of lncRNA‐ZEB2‐AS1 in breast cancer specimens and cells was related to clinicopathologic features and decreased survival of breast cancer patients

**Figure 1:**
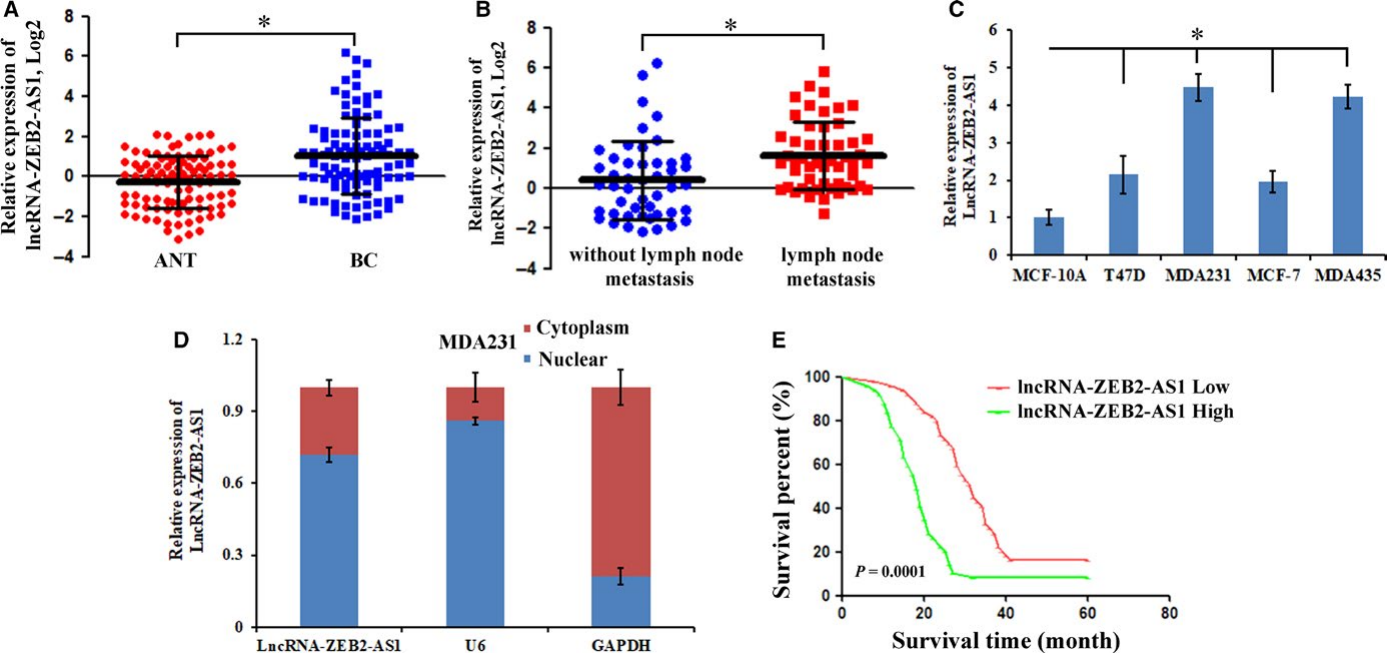
The differentially expressed of lncRNA‐ZEB2‐AS1 in breast cancer specimens and cells. A, Expression of lncRNA‐ZEB2‐AS1 in 98 pairs of BC and ANT specimens. B, Differentially expressed lncRNA‐ZEB2‐AS1 in breast cancer specimens with and without lymph node metastasis. C, LncRNA‐ZEB2‐AS1 detected in MCF‐7, T47D, MDA231, MDA435 and MCF‐10A cells. D, LncRNA‐ZEB2‐AS1 localization in the nucleus and cytoplasm was tested. E, Kaplan‐Meier method analysis (log‐rank test) was carried out for the verification of relationship between abnormal lncRNA‐ZEB2‐AS1 expression and poor prognosis (*, *P < *0.05).

To determine the biological function of lncRNA‐ZEB2‐AS1 in the tumorigenesis of triple‐negative breast cancer, we detected lncRNA‐ZEB2‐AS1 expression levels in 98 paired of BC specimens and ANT specimens by qRT‐PCR. The results revealed that lncRNA‐ZEB2‐AS1 was more markedly up‐regulated in breast cancer specimens than paired ANTs (Figure 1A). We further detected expressed lncRNA‐ZEB2‐AS1 with or without lymph node metastasis in breast cancer specimens (Figure 1B). Meanwhile, we measured the expression level of lncRNA‐ZEB2‐AS1 in breast cancer cells (T47D, MDA231, MCF‐7, and MDA435) and normal mammary epithelial cells (MCF‐10A). The data revealed that lncRNA‐ZEB2‐AS1 expression was markedly up‐regulated in breast cancer cells compared with MCF‐10A cells. The expression levels of highly invasive MDA231 cells and MDA435 were higher lncRNA‐ZEB2‐AS1, and those of invasive cells T47D and MCF‐7 were lower than lncRNA‐ZEB2‐AS1 (Figure 1C). LncRNA is considered to have different biological functions in different locations of the cells. Meanwhile, we first detected the distribution of lncRNA‐ZEB2‐AS1 in the MDA231 cells. LncRNA‐ZEB2‐AS1 was mainly located in the nucleus was minimally expressed in the cytoplasm (Figure 1D). Additionally, breast cancer patients with high lncRNA‐ZEB2‐AS1 levels have shorter survival rates than those with low levels (Figure 1E). The relationship between lncRNA‐ZEB2‐AS1 expression level and the clinicopathologic features of breast cancer patients was evaluated. LncRNA‐ZEB2‐AS1 expression is highly associated with tumor differentiation, lymph node metastasis, and distant metastasis in breast cancer but not associated with age or tumor size (Table 1). The results indicated that lncRNA‐ZEB2‐AS1 plays an oncogenic role and is associated with clinicopathologic features and decreased survival of breast cancer patients.

**Table 1:** Correlation between lncRNA‐ZEB2‐AS1 expression and clinicopathological features of breast cancer patients

Characteristics	Total (n = 98)	ZEB2‐AS1 expression		*P* value
		Low (n = 49)	High (n = 49)	
Age (years)				
≤50	51	26	25	0.500
≥51	47	23	24	
Tumor size (cm)				
≤5 cm	42	19	23	0.270
>5 cm	56	30	26	
Tumor differentiation				
I	21	16	5	0.013
II	48	23	25	
III	29	10	19	
Lymph node metastasis				
Yes	51	18	33	0.002
No	47	31	16	
Distant metastasis				
Yes	45	16	29	0.007
No	53	33	20	

### 3.2 Knockdown of lncRNA‐ZEB2‐AS1 suppressed the proliferation and invasion of MDA231 cells

**Figure 2:**
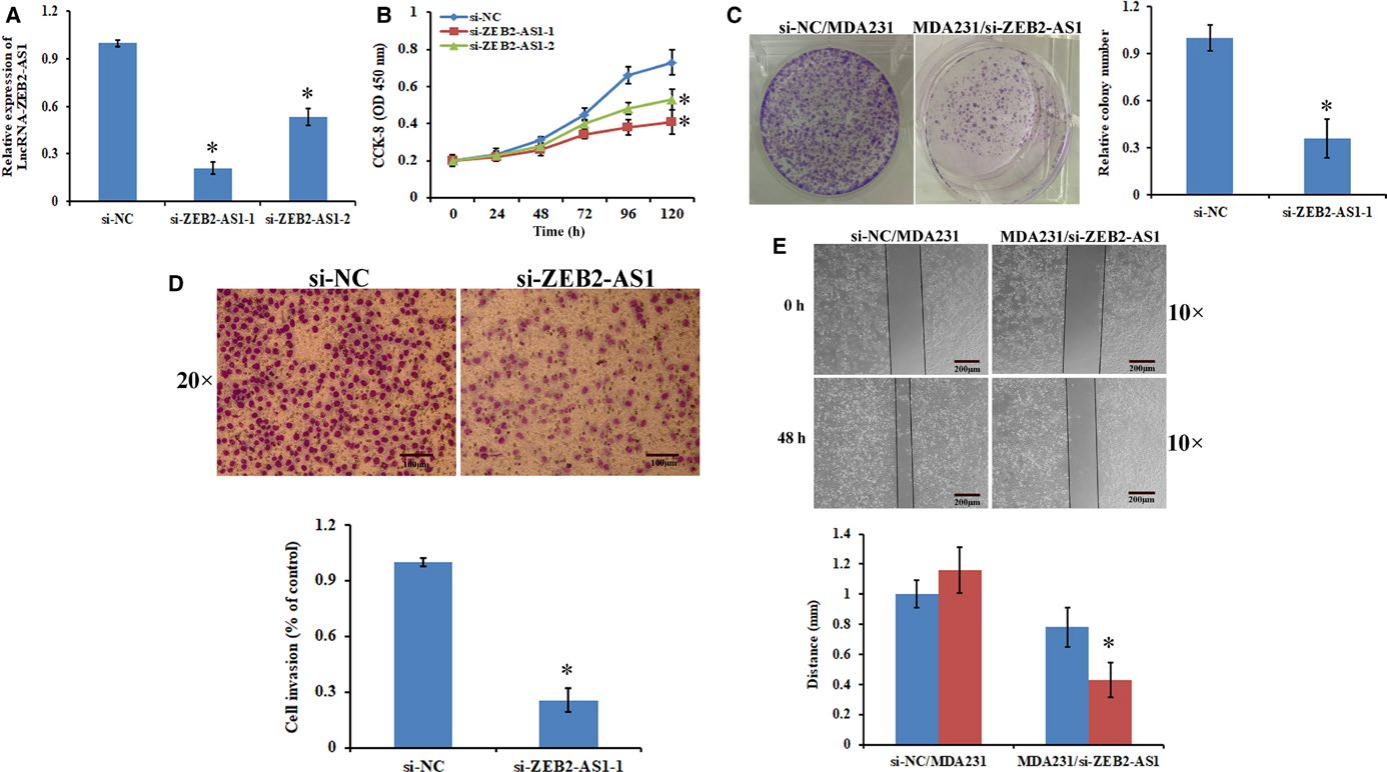
Knockdown of lncRNA‐ZEB2‐AS1 suppressed the proliferation and invasion of MDA231 cells. A, Depletion of lncRNA‐ZEB2‐AS1 in MDA231 cells by lncRNA‐ZEB1‐AS1‐RNAi. B, Cells proliferation activity was detected via CCK‐8 assays. C, Left: Colony formation assay results showed that the depletion of lncRNA ZEB2‐AS1 led to the remarkable decrease of MDA231/si‐ZEB2‐AS1 content. Right: Quantification of colony numbers. D, Left: Invasion of MDA231 cells analyzed with transwell chambers (scale bars = 100 μm). Right: Quantification of penetrating cells. E, Left: Wound healing assay (scale bars = 200 μm). Right: Migration distance (*, *P < *0.05).

The knockdown of lncRNA‐ZEB2‐AS1 in breast cancer cells implies that lncRNA‐ZEB2‐AS1 have important roles in breast cancer progression. To further investigate whether lncRNA‐ZEB2‐AS1 is connected to the occurrence of breast cancer, we researched the function of lncRNA‐ZEB2‐AS1 in vitro. As shown in Figure 2A, lncRNA‐ZEB2‐AS1‐RNAi‐1 (si‐ZEB2‐AS1) showed better interference efficiency than the negative control group (si‐NC). Thus, was selected for further research. In this research, the ability of cell proliferation was examined via CCK‐8 assays after the knockdown of lncRNA‐ZEB2‐AS1 in the MDA231 cells. The results revealed that the rate MDA231 cell proliferation was considerably reduced after the knockdown of lncRNA‐ZEB2‐AS1 (Figure 2B). Moreover, colonies formed by MDA231 cells were reduced (Figure 2C). To investigate the mechanisms underlying invasion suppression after reduction of lncRNA‐ZEB2‐AS1, we further tested the effect of lncRNA‐ZEB2‐AS1 on the invasion and migration ability of the MDA231 cells. The assay results of transwell and wound healing showed that the migration and invasion of MDA231 cells can be inhibited by reducing lncRNA‐ZEB2‐AS1 (Figure 2D,E). Overall, these results suggested that the decrease of lncRNA‐ZEB2‐AS1 expression led to the suppression of the proliferation and invasion of MDA231 cells.

### 3.3 Knockdown of lncRNA‐ZEB2‐AS1 led to the suppression of the tumorigenesis and metastasis of MDA231 cells in SCID mice

**Figure 3:**
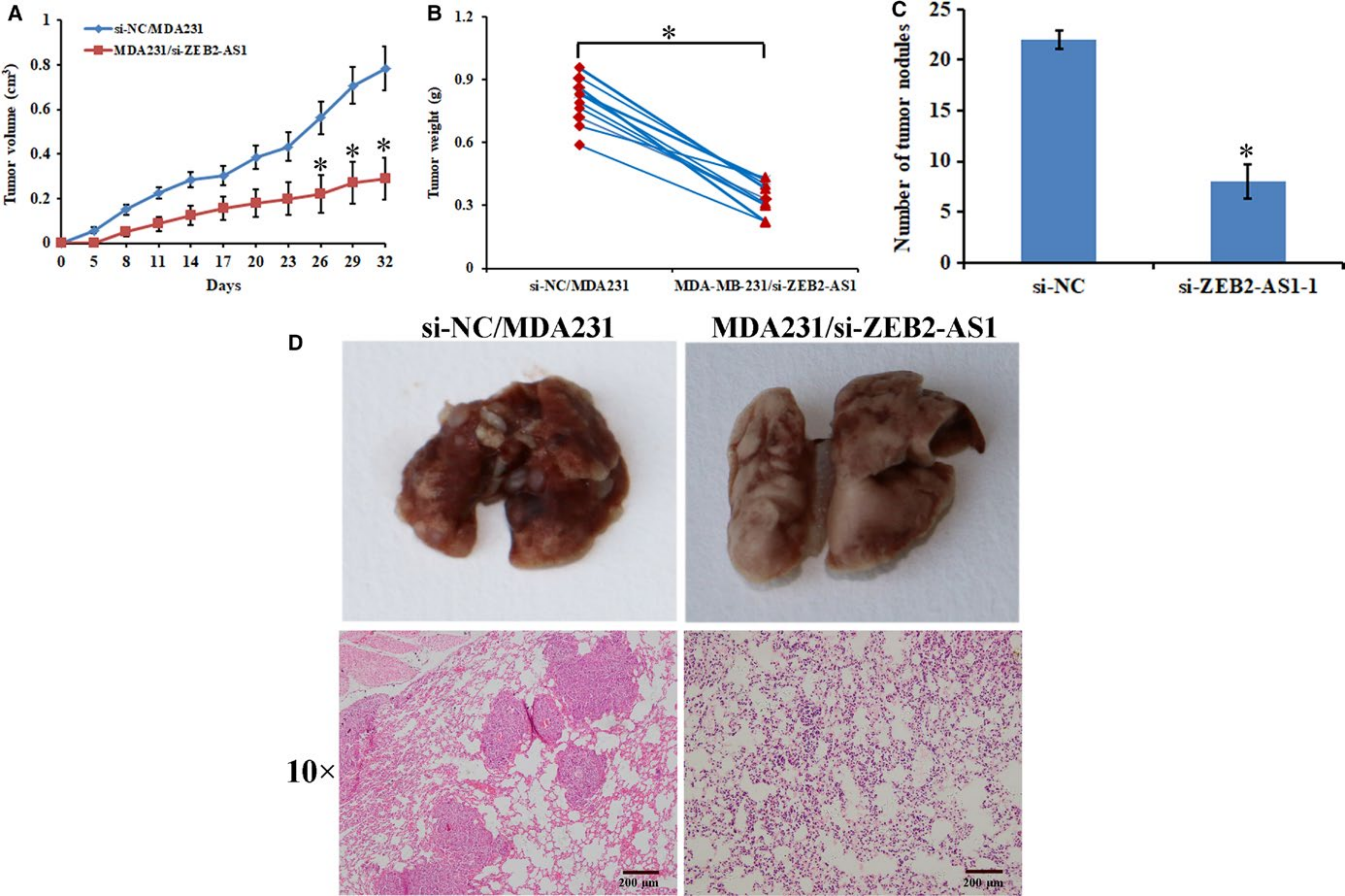
Knockdown of lncRNA‐ZEB2‐AS1 suppressed the tumorigenesis and metastasis of MDA231 cells in SCID mice. A, Tumor volume growth curves in SCID mice. B, Tumor weight at the end point. C, Number of tumor nodules at the end point. D, Pulmonary metastasis and HE staining pulmonary metastasis of SCID mice (scale bars = 200 μm) (*, *P < *0.05).

We further evaluated whether knocking down lncRNA‐ZEB2‐AS1 has an effect on the tumorigenesis and metastasis of MDA231 cells in SCID mice. We recorded changes in the volumes of MDA231/si‐ZEB2‐AS1 and si‐NC/MDA231 group cells during tumorigenesis. Compared with si‐NC/MDA231 group, MDA231/si‐ZEB2‐AS1 group grew more slowly. The final tumor volume in the MDA231/si‐ZEB2‐AS1 group was smaller than that in the si‐NC/MDA231 group (Figure 3A). Meanwhile, the final tumor weight in the former was lighter than that in the si‐NC/MDA231 group (Figure 3B). Changes in the metastasis capabilities of the MDA231 cells were detected after the stable transfection of si‐ZEB2‐AS1 and si‐NC plasmids in the SCID model. MDA231/si‐ZEB2‐AS1 group showed significantly reduced tumor nodules (Figure 3C). The H&E stains revealed that the MDA231/si‐ZEB2‐AS1 group had significantly reduced pulmonary metastatic foci (Figure 3D).

### 3.4 Knockdown of lncRNA‐ZEB2‐AS1 led to the down‐regulation of ZEB2 expression through PI3K/Akt/GSK3β/Zeb2 signaling pathway and inhibited EMT

**Figure 4:**
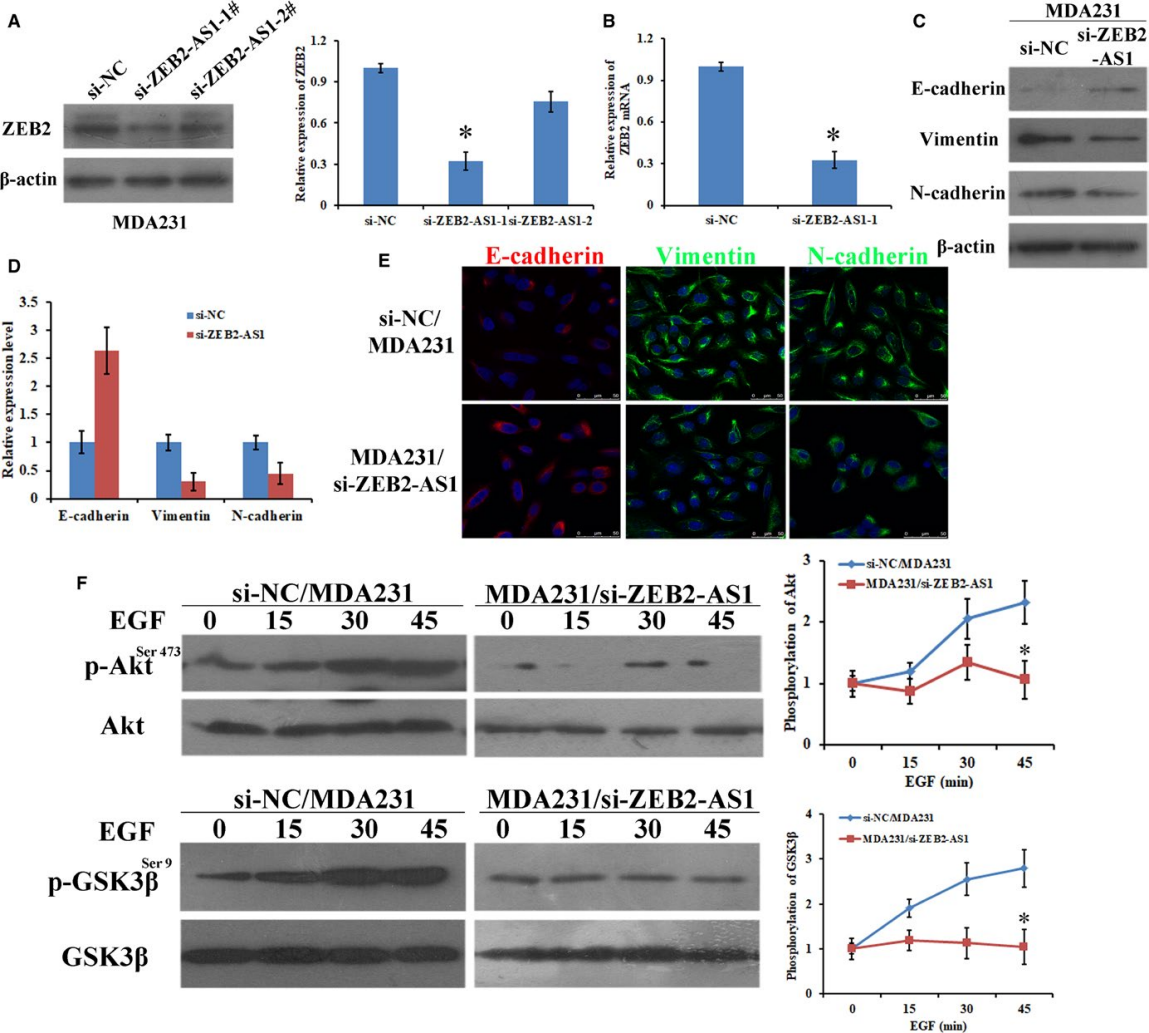
Knockdown of lncRNA‐ZEB2‐AS1 down‐regulated ZEB2 expression through PI3K/Akt/GSK3β/Zeb2 signaling pathway and inhibited EMT. A, Left: ZEB2 expression was tested using western blot. Right: quantification of ZEB2 protein. B, The expression of ZEB2 mRNA in MDA231 cells. C, The expression of EMT related markers was examined in MDA231 cells. D, Quantification of EMT related markers. E, Immunofluorescence stains (scale bars = 50 μm). F, Phosphorylated Akt^Ser473^ and GSK3β^Ser9^ in EGF‐induced MDA231 cells were examined (*, *P < *0.05).

The upstream antisense transcription may affect corresponding gene expression.^19^ LncRNA‐ZEB2‐AS1 is an antisense lncRNA. We hypothesized that the antisense transcript lncRNA‐ZEB2‐AS1 modulates ZEB2 in breast cancer. LncRNA‐ZEB2‐AS1 regulated the ZEB2 expression in the MDA231 cells. Then, we monitored the MDA231/si‐ZEB2‐AS1 cells to observe the effects of lncRNA‐ZEB2‐AS1 on ZEB2. ZEB2 was remarkably decreased in the MDA231/si‐ZEB2‐AS1 cells (Figure 4A). We then tested the expression level of ZEB2 mRNA. A remarkable positive correlation between LncRNA‐ZEB2‐AS1 and ZEB2 mRNA was observed at the protein level (Figure 4B). Given that ZEB2 is a crucial transcription factor modulating EMT in different malignant tumors, changes in the expression of EMT‐related markers in the MDA231/si‐ZEB2‐AS1 cells and si‐NC/MDA231 cells were investigated. Meanwhile, mesenchymal markers and epithelial markers were decreased and increased, respectively, when LncRNA‐ZEB2‐AS1 knocked down, suggesting that knocking down lncRNA‐ZEB2‐AS1 results in the inhibition of the EMT of breast cancer (Figure 4C,D). These results were supported by the EMT‐related markers detected by immunofluorescence (Figure 4E). Subsequently, we attempted to determine the important lncRNA‐ZEB2‐AS1 mechanisms that inhibit the EMT of the MDA231 cells. We discovered that the PI3K/Akt/GSK3β/Zeb2 signaling pathway has a pivotal role in the regulation of the EMT of the MDA231 cells. The phosphorylation levels of protein that played a key function in the PI3K/Akt/GSK3β/Zeb2 signaling pathway was tested by Western blot. Our data showed that the phosphorylation levels of GSK3β and Akt in MDA231/si‐ZEB2‐AS1 cells were considerably depressed when lncRNA‐ZEB2‐AS1 expression was knocked down (Figure 4F). All the results validated that knockdown of lncRNA‐ZEB2‐AS1 leads to the down‐regulation of ZEB2 expression and subsequently to the suppression of EMT through the PI3K/Akt/GSK3β/Zeb2 signaling pathway.

### 3.5 Reduction of lncRNA‐ZEB2‐AS1 suppressed EGF‐induced F‐actin polymerization in MDA231 cells

**Figure 5:**
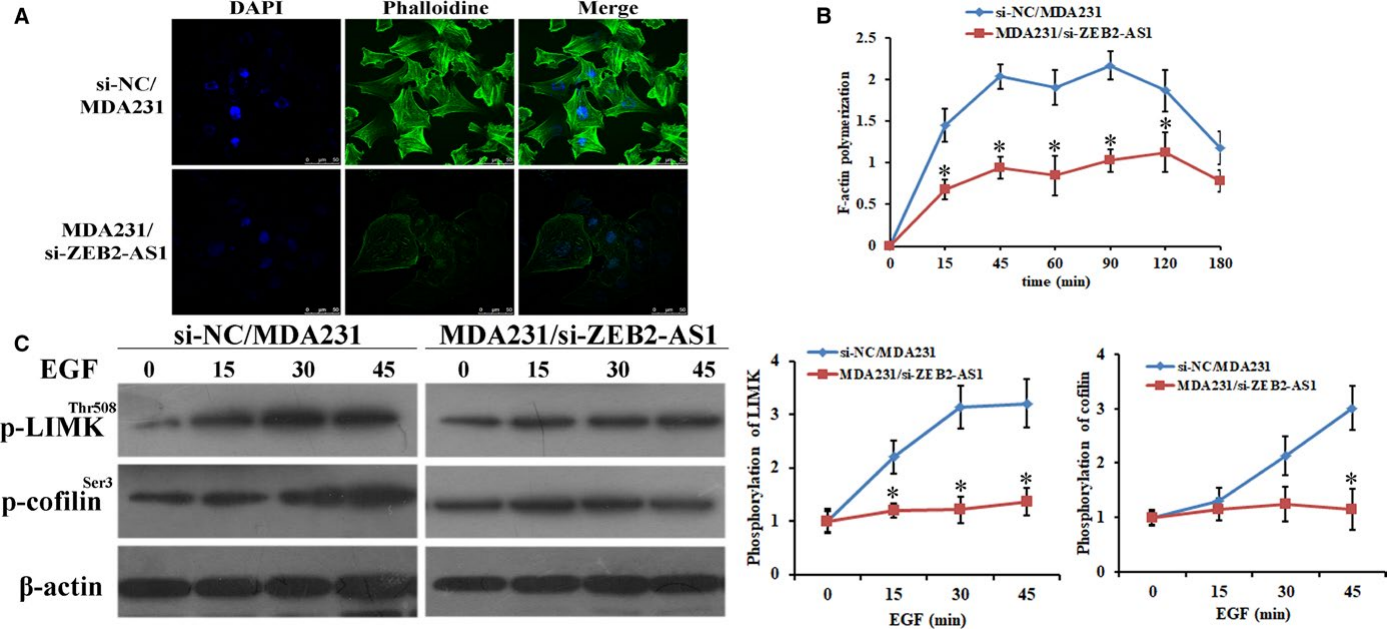
Reduction of lncRNA‐ZEB2‐AS1 suppressed EGF‐induced F‐actin polymerization in MDA231 cells. A, Cytoskeleton rearrangement in the MDA231 cells was shown by fluorescence assay (scale bars = 50 μm). B, F‐actin polymerization in the MDA231 cells under different periods of stimulation with EGF. C, P‐LIMK^Thr508 ^and p‐cofilin^Ser3^ in MDA231 cells under EGF stimulation (*, *P < *0.05).

F‐actin polymerization is an important mechanism for the invasion and metastasis of cancer cells. To confirm the putative that the depression of lncRNA‐ZEB2‐AS1 could suppress EGF‐induced F‐actin polymerization of MDA231 cells, we performed the F‐actin polymerization assay to detect the effect of lncRNA‐ZEB2‐AS1 reduction on the invasion and metastasis capacity of MDA231 cells with EGF stimulation. The data showed that EGF led to short actin polymerization in si‐NC/MDA231 cells and was notably decreased in the MDA231/si‐ZEB2‐AS1 cells, indicating that lncRNA‐ZEB2‐AS1 played a vital function in modulating cytoskeleton rearrangement by EGF stimulation (Figure 5A,B). Meanwhile, LIMK and cofilin are two vital proteins related to the regulation of F‐actin polymerization.^20^ We detected the phosphorylation status of LIMK and cofilin, to confirm whether lncRNA‐ZEB2‐AS1 inhibits EGF‐induced rearrangement of cytoskeletons in the MDA231 cells (Figure 5C). As shown in Figure 5C, the reduction of lncRNA‐ZEB2‐AS1 expression depressed the phosphorylation levels of LIMK and cofilin, and the cytoskeleton was abolished. These results indicated that reduction of lncRNA‐ZEB2‐AS1 suppressed EGF‐induced F‐actin polymerization in MDA231 cells.

### 3.6 Clinical relevance of lncRNA‐ZEB2‐AS1 between ZEB2 and EMT related markers in human breast cancer

**Figure 6:**
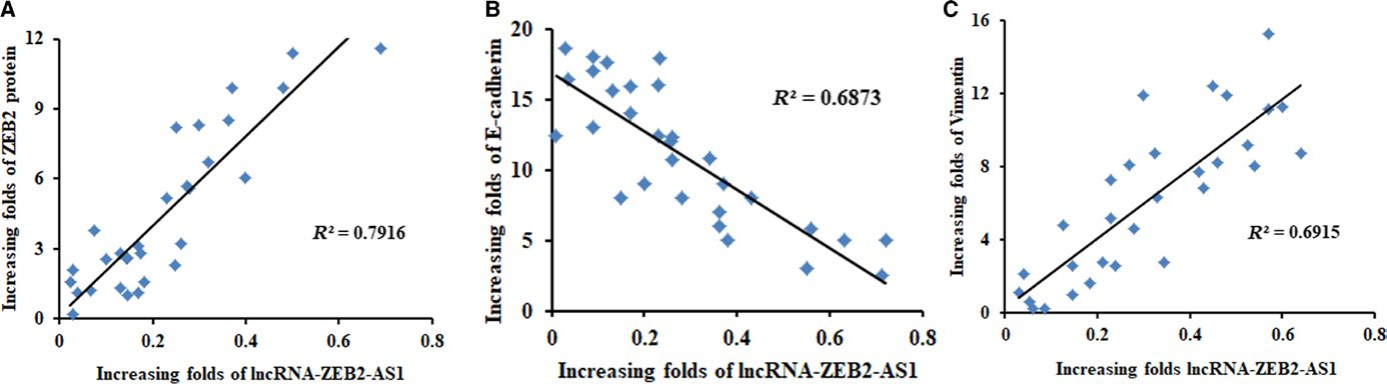
Clinical relevance of lncRNA‐ZEB2‐AS1 between ZEB2 and EMT‐related marker in human breast cancer. A, Correlation between lncRNA‐ZEB2‐AS1 and ZEB2. B, Correlation between lncRNA‐ZEB2‐AS1 and E‐cadherin, C, correlation between lncRNA‐ZEB2‐AS1 and vimentin were all confirmed through clinical correlation coefficient analysis

In order to further indicate the clinically relevant by which lncRNA‐ZEB2‐AS1 modulated ZEB2 and EMT related markers. We detected the lncRNA‐ZEB2‐AS1 expression and its association with the expression levels of ZEB2 in the clinical tissues of breast cancer paired with breast cancer specimens and ANT specimens. The relationship between the expression levels of EMT related markers and lncRNA‐ZEB2‐AS1 was investigated. In Figure 6, a positive correlation among lncRNA‐ZEB2‐AS1, ZEB2, and vimentin in 30 tested clinical specimens was observed, whereas lncRNA‐ZEB2‐AS1 was negatively correlated with E‐cadherin. All the results suggested that lncRNA‐ZEB2‐AS1 in the ZEB2 and EMT‐related markers is clinically relevant in breast cancer.

## 4 DISCUSSION

An increasing number of studies have reported that LncRNA considerably affects cellular processes, such invasion, metastasis, metabolism, and apoptosis.^21^, ^22^ LncRNAs monitor carcinogenesis principally via modulating the expression levels of oncogenes or tumor suppressors.^19^ Many studies revealed that LncRNA‐ZEB2‐AS1 decreases tumor growth and metastasis in HCC.^23^ LncRNA‐ZEB2‐AS1 is a newly discovered lncRNA, whose clinical meaning, biological roles, and explicit mechanism are poorly understood in breast cancer. We first confirmed that lncRNA‐ZEB2‐AS1 is abnormally expressed in breast cancer specimens, and this abnormality is related to the overall survival and clinicopathologic features of patients with breast cancer. Mechanistically, lncRNA‐ZEB2‐AS1 affects the proliferation, metastasis, and EMT of MDA231 cells.

ZEB1 and ZEB2 cardinal proteins of the ZEB family. These proteins promote EMT in cancer. Transcription factor attributing to especial Zinc finger protein families, usually located in the downstream of some signaling pathways executing EMT process in normal and pathological conditions.^24^ Previous discuss have indicated that there is ectopic of ZEB in tumor and is associated with poor prognosis in patients.^25^ ZEB1 is a novel protein and pivotal transcription factor in EMT progress. ZEB2 protein might have a tumor promoter role because it controls cell cycle progression and cell differentiation.^26^ Wang et al found that E2F1 promoted EMT by modulating the promoter region of ZEB2 in NSCLC.^27^ ZEB2 is a transcription factor that participates in adjusting different biological activities, including the inhibition of apoptosis of vascular endothelial cells via the MAPK pathway activation.^28^ Meanwhile, the mechanisms by which lncRNA‐ZEB2‐AS1 regulates EMT in breast cancer remain unknown. In this study, we found that knocking down lncRNA‐ZEB2‐AS1 results in the down‐regulation of ZEB2 expression through the PI3K/Akt/GSK3β/Zeb2 signaling pathway, inactivation of AKT and GSK3β phosphorylation, inhibition of EMT in breast cancer.

Distant metastasis in early phases of development is a typical biological characteristic of cancer cells, and F‐actin polymerization is considered to be an early, pivotal step for invasion and metastasis. The number arrays of actin binding proteins are the modulators of F‐actin polymerization and lamellipodium formation.^29^ LIMK and cofilin are essential controllers.^30^, ^31^ B. Kalyanaraman et al recent research also found that cancer cell invasion needs essential regulations of cell cytoskeleton, cell‐to‐matrix and cellular adhesions.^32^ Wang et al found that migration and invasion inhibitory proteins inhibit endometrial carcinoma migration via the cytoskeleton reorganization when the number of lamellipodia are markedly reduced.^33^ It is still an unknown molecular mechanism that lncRNA‐ZEB2‐AS1 modulates F‐actin polymerization of MDA231 cells. In the current research, we detected the rearrangement of cytoskeletal protein when lncRNA‐ZEB2‐AS1 was abrogated in breast cancer cells. Meanwhile, the phosphorylation levels of LIMK and cofilin were decreased after lncRNA‐ZEB2‐AS1 was knocked down, verifying that lncRNA‐ZEB2‐AS1 was related to F‐actin polymerization in MDA231 cells.

Collectively, our results showed that lncRNA‐ZEB2‐AS1/PI3K/Akt/GSK3β/Zeb2 axis facilitates tumor progression and is a potential prevention target in breast cancer.

## CONFLICT OF INTEREST

The authors confirm that there are no conflicts of interest.

## AUTHOR CONTRIBUTIONS

Guoxin Zhang, Hongli Li, Zhiyi Yang, and Yuanyuan Liu performed experiments and wrote the manuscript. Zhaoyan Wang and Yuling Yang analyzed data. Ruimei Sun, Peirui Li and Chonggao Yin assisted with the design of experiments.
