# Supplementary material for: Long non‐coding RNA ZEB2‐AS1 promotes the proliferation, metastasis and epithelial mesenchymal transition in triple‐negative breast cancer by epigenetically activating ZEB2
Source: J Cell Mol Med. 2019 Mar 1;23(5):3271–9. doi: 10.1111/jcmm.14213 (PMC6484319; doi:10.1111/jcmm.14213)
Supplement: Supplementary file 1 [file JCMM-23-3271-s001.docx]

**Supplementary Table S1** Primers used for quantitative real-time PCR

| ZEB2-AS1 Forward 5’-CATGAAGAAGCCGCGAAGTG-3’  Reverse 5 -CGTTTTCCGCCCTGTACTCT-3’  ZEB2 Forward 5’-AAGGAGCAGGTAATCGCAAG-3’  Reverse 5’-TTTGGGCACTCGTAAGGTTT-3’  E-cadherin Forward 5’-GGTGCTCTTCCAGGAACCTC -3’  Reverse 5’-GAAACTCTCTCGGTCCAGCC-3’  Vimentin Forward 5’-CGGGAGAAATTGCAGGAGGA-3’  Reverse 5’-AAGGTCAAGACGTGCCAGAG-3’  GAPDH Forward 5’-GCACCGTCAAGGCTGAGAAC-3’  Reverse 5’-TGGTGAAGACGCCAGTGGA-3’ |
| --- |

**Supplementary Table S2** Sequences of siRNAs

| si-ZEB2-AS1-1 5’-GCCTCGAGGATTAGTTTAAAC-3’  Si-ZEB2-AS1-2 5’-GCTCTACTAAATGATCGTATC-3’ |
| --- |
